# Supplementary material for: Characterization of Clinical Features of Hospitalized Patients Due to the SARS-CoV-2 Infection in the Absence of Comorbidities Regarding the Sex: An Epidemiological Study of the First Year of the Pandemic in Brazil
Source: Int J Environ Res Public Health. 2022 Jul 22;19(15):8895. doi: 10.3390/ijerph19158895 (PMC9331852; doi:10.3390/ijerph19158895)
Supplement: Supplementary file 1 [file ijerph-19-08895-s001.zip › ijerph-1756388-supplementary.pdf]

Title: Characterization of Clinical Features of Hospitalized Patients due to the SARS-CoV-2 Infection in the Absence of Comorbidities Regarding the Sex: An Epidemiological Study of the First Year of the Pandemic in Brazil

Short title: Sex and COVID-19

Table S1. Distribution of patients hospitalized with the severe acute respiratory syndrome (SARS) due to Coronavirus Disease (COVID)-19 in Brazil according to patients′ sex in the federative unit of residence.

State and Federal District	Male	Female	Total
Acre	821 (0.4%)	468 (0.3%)	1289 (0.4%)
Alagoas	2161 (1.0%)	1364 (1.1%)	3525 (1.0%)
Amazonas	8833 (4.1%)	5983 (4.9%)	14,816 (4.4%)
Amapá	785 (0.4%)	514 (0.4%)	1299 (0.4%)
Bahia	6612 (3.1%)	3498 (2.8%)	10,110 (3.0%)
Ceará	12547 (3.7%)	4626 (3.8%)	12,547 (3.7%)
Federal District	4864 (2.3%)	1820 (1.5%)	6684 (2.0%)
Espírito Santo	1814 (0.9%)	1041 (0.8%)	2855 (0.8%)
Goiás	7967 (3.7%)	4603 (3.7%)	12,570 (3.7%)
Maranhão	2583 (1.2%)	1548 (1.3%)	4131 (1.2%)
Minas Gerais	14,640 (6.9%)	8616 (7.0%)	23,256 (6.9%)
Mato Grosso do Sul	3471 (1.6%)	2101 (1.7%)	5572 (1.7%)
Mato Grosso	8529 (4.0%)	6436 (5.2%)	14,965 (4.4%)
Pará	7896 (3.7%)	4704 (3.8%)	12,600 (3.7%)
Paraíba	2905 (1.4%)	1865 (1.5%)	4770 (1.4%)
Pernambuco	8196 (3.8%)	4899 (4.0%)	13,095 (3.9%)
Piauí	2683 (1.3%)	1432 (1.2%)	4115 (1.2%)
Paraná	11,507 (5.4%)	6679 (5.4%)	18,186 (5.4%)
Rio de Janeiro	19,206 (9.0%)	10,706 (8.7%)	29,912 (8.9%)
Rio Grande do Norte	1713 (0.8%)	844 (0.7%)	2557 (0.8%)
Rondônia	2437 (1.1%)	1312 (1.1%)	3749 (1.1%)
Roraima	512 (0.2%)	286 (0.2%)	798 (0.2%)
Rio Grande do Sul	10,487 (4.9%)	6153 (5.0%)	16,640 (4.9%)
Santa Catarina	8513 (4.0%)	4591 (3.7%)	13,104 (3.9%)
Sergipe	2522 (1.2%)	1423 (1.2%)	3945 (1.2%)
São Paulo	62,174 (29.2%)	35,068 (28.4%)	97,242 (28.9%)
Tocantins	1399 (0.7%)	732 (0.6%)	2131 (0.6%)
Total	213,151 (100.0%)	123,312 (100.0%)	336,463 (100.0%)

We presented the data using absolute (N) and relative (%) frequencies.

Table S2. The distribution of patients hospitalized with the severe acute respiratory syndrome (SARS) due to Coronavirus Disease (COVID)-19 in Brazil according to sex, weeks of disease notification, and the start of clinical signs.

Weeks	Week of notification			Week of first clinical signs		
	Male	Female	Total	Male	Female	Total
8	1 (100.0%)	0 (0.0%)	1	10 (55.6%)	8 (44.4%)	18
9	4 (50.0%)	4 (50.0%)	8	13 (40.6%)	19 (59.4%)	32
10	1 (25.0%)	3 (75.0%)	4	50 (61.7%)	31 (38.3%)	81
11	18 (60.0%)	12 (40.0%)	30	257 (63.6%)	147 (36.4%)	404
12	214 (60.3%)	141 (39.7%)	355	869 (64.3%)	483 (35.7%)	1352
13	750 (63.8%)	425 (36.2%)	1175	1268 (66.5%)	638 (33.5%)	1906
14	1274 (65.0%)	687 (35.0%)	1961	1651 (65.1%)	887 (34.9%)	2538
15	1517 (65.8%)	789 (34.2%)	2306	2452 (66.3%)	1244 (33.7%)	3696
16	2359 (65.0%)	1270 (35.0%)	3629	3488 (65.8%)	1811 (34.2%)	5299
17	3165 (65.0%)	1703 (35.0%)	4868	4608 (66.3%)	2340 (33.7%)	6948
18	4005 (65.2%)	2135 (34.8%)	6140	5099 (64.9%)	2752 (35.1%)	7851
19	4516 (64.6%)	2473 (35.4%)	6989	5142 (64.1%)	2875 (35.9%)	8017
20	5005 (65.4%)	2648 (34.6%)	7653	5528 (65.2%)	2957 (34.8%)	8485
21	5088 (64.7%)	2778 (35.3%)	7866	4737 (63.6%)	2716 (36.4%)	7453
22	4584 (63.3%)	2657 (36.7%)	7241	4185 (62.1%)	2557 (37.9%)	6742
23	4193 (63.2%)	2446 (36.8%)	6639	4712 (62.4%)	2844 (37.6%)	7556
24	4415 (63.6%)	2530 (36.4%)	6945	4674 (62.7%)	2779 (37.3%)	7453
25	4851 (61.9%)	2987 (38.1%)	7838	5158 (62.1%)	3144 (37.9%)	8302
26	4840 (62.9%)	2858 (37.1%)	7698	4520 (62.8%)	2679 (37.2%)	7199
27	4882 (63.0%)	2871 (37.0%)	7753	4945 (62.1%)	3014 (37.9%)	7959
28	4932 (62.5%)	2960 (37.5%)	7892	4818 (64.7%)	2625 (35.3%)	7443
29	4654 (64.3%)	2583 (35.7%)	7228	4282 (64.6%)	2350 (35.4%)	6632
30	4692 (63.6%)	2680 (36.4%)	7372	3865 (62.5%)	2319 (37.5%)	6.184
31	4118 (63.6%)	2356 (36.4%)	6474	3574 (62.9%)	2104 (37.1%)	5678
32	3583 (63.8%)	2035 (36.2%)	5618	3255 (64.0%)	1834 (36.0%)	5089
33	3375 (63.2%)	1961 (36.8%)	5336	3313 (64.9%)	1791 (35.1%)	5104
34	3249 (64.4%)	1795 (35.6%)	5044	3026 (63.0%)	1780 (37.0%)	4806
35	3129 (63.2%)	1824 (36.8%)	4953	2779 (63.7%)	1551 (36.3%)	4271
36	3058 (64.2%)	1704 (35.8%)	4762	2779 (63.9%)	1573 (36.1%)	4352
37	2705 (62.7%)	1611 (37.3%)	4316	2416 (61.9%)	1490 (38.1%)	3906
38	2626 (62.5%)	1573 (37.5%)	4199	2083 (61.9%)	1280 (38.1%)	3363
39	2390 (62.6%)	1428 (37.4%)	3818	2052 (61.6%)	1277 (38.4%)	3329
40	2196 (62.2%)	1336 (37.8%)	3532	1997 (62.4%)	1204 (37.6%)	3201
41	2019 (61.7%)	1253 (38.3%)	3272	1897 (61.8%)	1171 (38.2%)	3068
42	1779 (62.1%)	1085 (37.9%)	2864	1804 (63.5%)	1038 (36.5%)	2842
43	1886 (62.7%)	1122 (37.3%)	3008	1909 (64.2%)	1064 (35.8%)	2973
44	1902 (63.1%)	1110 (36.9%)	3012	1901 (63.5%)	1094 (36.5%)	2995
45	1931 (64.2%)	1079 (35.8%)	3010	2715 (64.2%)	1515 (35.8%)	4230
46	2392 (64.1%)	1337 (35.9%)	3729	3144 (64.5%)	1733 (35.5%)	4877
47	3036 (63.2%)	1767 (36.8%)	4803	3744 (63.9%)	2111 (36.1%)	5855
48	3562 (63.7%)	2032 (36.3%)	5594	3835 (64.1%)	2146 (35.9%)	5981
49	3907 (64.3%)	2165 (35.7%)	6072	4206 (63.4%)	2429 (36.6%)	6635
50	3972 (63.5%)	2285 (36.5%)	6257	3732 (62.2%)	2268 (37.8%)	6000
51	3824 (62.9%)	2259 (37.1%)	6083	3405 (62.5%)	2042 (37.5%)	5447
52	3347 (63.0%)	1967 (37.0%)	5314	3828 (63.0%)	2251 (37.0%)	6079

53	3558 (62.6%)	2130 (37.4%)	5688	4349 (60.8%)	2802 (39.2%)	7151
54	4354 (60.8%)	2809 (39.2%)	7163	4597 (61.8%)	2845 (38.2%)	7442
55	4535 (61.9%)	2789 (38.1%)	7324	4551 (62.5%)	2726 (37.5%)	7277
56	4436 (62.5%)	2664 (37.5%)	7100	3991 (62.0%)	2448 (38.0%)	6439
57	3951 (62.6%)	2365 (37.4%)	6316	3772 (62.7%)	2247 (37.3%)	6019
58	4143 (63.3%)	2433 (37.0%)	6576	4706 (62.2%)	2857 (37.8%)	7563
59	4389 (62.4%)	2645 (37.6%)	7034	5541 (63.7%)	3164 (36.3%)	8705
60	4974 (62.7%)	2958 (37.3%)	7932	7197 (63.9%)	4073 (36.1%)	11,270
61	6491 (63.1%)	3793 (36.9%)	10,284	7610 (63.7%)	4331 (36.3%)	11,941
62	7770 (63.3%)	4508 (36.7%)	12,278	10,021 (63.6%)	5728 (36.4%)	15,749
63	8934 (63.4%)	5164 (36.6%)	14,098	8725 (63.2%)	5077 (36.8%)	13,802
64	9297 (63.5%)	5345 (36.5%)	14,642	6259 (63.3%)	3626 (36.7%)	9885
65	8099 (63.8%)	4602 (36.2%)	12,701	1980 (60.5%)	1294 (39.5%)	3274
66	4176 (64.1%)	2334 (35.9%)	6510	186 (59.2%)	128 (40.8%)	314
67	107 (68.6%)	49 (31.4%)	156	0 (0.0%)	1	1
Total	213,151 (63.4%)	123,312 (36.6%)	336,463	213,151 (63.4%)	123,312 (36.6%)	336,463

We presented the data using absolute (N) and relative (%) frequencies.

Table S3. Characterization of patients hospitalized with the severe acute respiratory syndrome (SARS) due to Coronavirus Disease (COVID)-19.

**Patients' features**	**Marker**	**N (%)**
Sex	Male	213,151 (63.4%)
	Female	123,312 (36.6%)
Age	<1 year old	3625 (1.1%)
	1-12 years old	5638 (1.7%)
	13-24 years old	9774 (2.9%)
	25-60 years old	203,144 (60.4%)
	61-72 years old	61,803 (18.4%)
	+85 years old	39,639 (11.8%)
Race	White	126,871 (47.8%)
	Black	12,271 (4.6%)
	Asian	3458 (1.3%)
	Pardos (Multiracial background)	121,505 (45.8%)
	Indigenous	1234 (0.4%)
Schooling	Illiterate	6038 (4.9%)
	Elementary school (1st stage)	24,076 (19.7%)
	Elementary school (2nd stage)	19,739 (16.1%)
	High school	44,857 (36.6%)
	Higher education	23,477 (19.2%)
	Not applicable	4336 (3.5%)
Place of residence	Urban	276,515 (94.7%)
	Rural	14,572 (5.0%)
	Peri-urban	960 (0.3%)
Residence in Influenza outbreak area	Yes	64,192 (30.8%)
	No	144,462 (69.2%)
Presence of nosocomial infection	Yes	3375 (1.5%)
	No	221,342 (98.5%)
Clinical symptoms		
Fever	Yes	210,247 (74.2%)
Cough	Yes	234,488 (81.0%)
Sore throat	Yes	73,488 (31.2%)
Dyspnea	Yes	219,367 (77.0%)
Respiratory distress	Yes	179,547 (67.9%)
Peripheral arterial oxygen saturation <95%	Yes	174,584 (65.9%)
Diarrhea	Yes	45,518 (20.1%)
Vomit	Yes	25,426 (11.5%)
Abdominal pain	Yes	15,673 (9.9%)
Fatigue and asthenia	Yes	68,408 (39.6%)
Loss of smell	Yes	34,960 (21.1%)
Loss of taste	Yes	32,579 (19.9%)
Myalgia	Yes	36,584 (16.5%)
Headache	Yes	36,591 (16.5%)
Coryza	Yes	9876 (4.4%)
Inappetence	Yes	6196 (2.8%)
Nasal obstruction	Yes	1543 (0.7%)
Vertigo	Yes	1291 (0.6%)
Prostration	Yes	3961 (1.8%)

Nausea	Yes	3797 (1.7%)
Malaise	Yes	3312 (1.5%)
Chills	Yes	2331 (1.0%)
Chest pain	Yes	6517 (2.9%)
Back pain	Yes	3277 (1.5%)
Arthralgia	Yes	1183 (0.5%)
Antiviral drug to treat flu symptoms	Yes	32,322 (14.2%)
	No	195,014 (85.8%)
Thorax X-ray	Normal	6320 (3.9%)
	Interstitial infiltrate	40,439 (24.8%)
	Consolidation	4426 (2.7%)
	Mixed	5873 (3.6%)
	Other findings	21,384 (13.1%)
	Not carried out	84,552 (51.9%)
Thorax high-resolution computerized tomography	Typical COVID-19	90,227 (65.9%)
	undetermined COVID-19	3454 (2.5%)
	Atypical COVID-19	1782 (1.3%)
	Negative for pneumonia	469 (0.3%)
	Not carried out	5142 (3.8%)
	Ignored	35,744 (26.1%)
Intensive care unit	Yes	75,465 (28.4%)
	No	190,458 (71.6%)
Use of ventilatory support	Invasive	37,920 (14.3%)
	Noninvasive	142,070 (53.7%)
	Not necessary	84,414 (31.9%)
Closing criterion	Laboratory	296,625 (92.4%)
	Epidemiological clinical	3436 (1.1%)
	Clinical	7952 (2.5%)
	Image clinical	12,856 (4.0%)
Outcome	Cure	206,810 (73.7%)
	Death	73,723 (26.3%)

Data presented as absolute (N) and relative (%) frequencies.

Table S4. Multivariate analysis determines death predictors in patients with Coronavirus Disease and morbidities in Brazil.

Death predictors [a]	B	SE	Wald	df	*P*	OR	95% CI	
							Lower	Higher
Sex (Male)	0.096	0.044	4.891	1	0.027	1.101	1.011	1.199
Age								
<1 year old			1.291.316	6	<0.001			
1-12 years old	-0.566	1.205	0.220	1	0.639	0.568	0.054	6.030
13-24 years old	1.035	1.106	0.875	1	0.349	2.815	0.322	24.595
25-60 years old	1.627	1.089	2.232	1	0.135	5.090	0.602	43.038
61-72 years old	2.759	1.090	6.410	1	0.011	15.778	1.865	133.492
73-85 years old	3.465	1.090	10.113	1	0.001	31.978	3.779	270.600
+85 years old	4.240	1.092	15.078	1	<0.001	69.385	8.164	589.705
Race								
White			115.881	4	<0.001			
Black	0.221	0.105	4.448	1	0.035	1.247	1.016	1.531
Asian	0.349	0.201	3.015	1	0.082	1.417	0.956	2.101
Pardos (Multiracial background)	0.461	0.045	103.179	1	<0.001	1.585	1.450	1.732
Indigenous	1.159	0.256	20.423	1	<0.001	3.186	1.927	5.266
Schooling								
Illiterate	0.726	0.105	47.751	1	<0.001	2.066	1.682	2.538
Elementary school (1st stage)	0.547	0.074	55.258	1	<0.001	1.728	1.496	1.997
Elementary school (2nd stage)	0.404	0.075	28.666	1	<0.001	1.497	1.292	1.736
High school	0.209	0.069	9.128	1	0.003	1.232	1.076	1.411
Higher education			78.960	4	<0.001			
Zone (Rural or peri-urban)	-0.179	0.093	3.665	1	0.056	0.836	0.696	1.004
Residence in the Influenza outbreak area	0.107	0.048	4.968	1	0.026	1.113	1.013	1.222
Clinical symptoms								
Dyspnea	0.208	0.053	15.498	1	<0.001	1.231	1.110	1.365
SpO_2	0.312	0.050	38.578	1	<0.001	1.367	1.238	1.508
Diarrhea	-0.112	0.056	3.986	1	0.046	0.894	0.800	0.998
Myalgia	-0.282	0.077	13.489	1	<0.001	0.754	0.648	0.877
Headache	-0.252	0.076	10.931	1	0.001	0.777	0.670	0.903
Inappetence	-0.270	0.145	3.502	1	0.061	0.763	0.575	1.013
Cyanosis	0.973	0.574	2.866	1	0.090	2.645	0.858	8.155
Nasal obstruction	-1.051	0.590	3.171	1	0.075	0.350	0.110	1.112
Nausea	-0.415	0.238	3.026	1	0.082	0.660	0.414	1.054
Flu vaccination	-0.337	0.049	47.211	1	<0.001	0.714	0.649	0.786
Need for ICU	1.121	0.049	527.915	1	<0.001	3.069	2.789	3.377
Need for ventilatory support								
Ventilatory support			1.212.079	2	<0.001			
Invasive	2.320	0.074	986.304	1	<0.001	10.174	8.803	11.759
Noninvasive	0.475	0.057	68.752	1	<0.001	1.609	1.438	1.800
Not needed								
Constant	-5.601	1.092	26.311	1	<0.001	0.004		

SE, standard error; df, degrees of freedom; OR, odds ratio; 95%CI, 95% confidence interval; ICU, Intensive care unit.

[a.] Variables inserted in step 1: sex, age, race, schooling, zone, residing in influenza syndrome outbreak area, presence of nosocomial infection, clinical signs (fever, cough, sore throat, dyspnea, respiratory distress, peripheral arterial oxygen saturation (SpO_2), diarrhea, vomit, abdominal pain, loss of smell, loss of taste, myalgia, headache, coryza, inappetence, cyanosis, nasal obstruction, vertigo, nausea, chills, sneeze, back pain, lower limb pain, flu vaccination, use of the antiviral drug to treat flu symptoms, need for intensive care treatment and need for ventilatory support.
